# Long-Term Trends in Unintentional Fall Mortality in China: A Population-Based Age-Period-Cohort Study

**DOI:** 10.3389/fpubh.2021.749295

**Published:** 2021-11-24

**Authors:** Zhenkun Wang, Youzhen Hu, Fang Peng

**Affiliations:** ^1^Outpatient Department, Tongji Hospital, Tongji Medical College, Huazhong University of Science and Technology, Wuhan, China; ^2^Department of Scientific Research, Tongji Hospital, Tongji Medical College, Huazhong University of Science and Technology, Wuhan, China; ^3^Department of Emergency, Tongji Hospital, Tongji Medical College, Huazhong University of Science and Technology, Wuhan, China

**Keywords:** unintentional falls, mortality trend risk, age-period-cohort (APC) analysis, China, injury

## Abstract

**Background:** Unintentional falls seriously threaten the life and health of people in China. This study aimed to assess the long-term trends of mortality from unintentional falls in China and to examine the age-, period-, and cohort-specific effects behind them.

**Methods:** This population-based multiyear cross-sectional study of Chinese people aged 0–84 years was a secondary analysis of the mortality data of fall injuries from 1990 to 2019, derived from the Global Burden of Disease Study 2019. Age-standardized mortality rates of unintentional falls by year, sex, and age group were used as the main outcomes and were analyzed within the age-period-cohort framework.

**Results:** Although the crude mortality rates of unintentional falls for men and women showed a significant upward trend, the age-standardized mortality rates for both sexes only increased slightly. The net drift of unintentional fall mortality was 0.13% (95% CI, −0.04 to 0.3%) per year for men and −0.71% (95% CI, −0.96 to −0.46%) per year for women. The local drift values for both sexes increased with age group. Significant age, cohort, and period effects were found behind the mortality trends of the unintentional falls for both sexes in China.

**Conclusions:** Unintentional falls are still a major public health problem that disproportionately threatens the lives of men and women in China. Efforts should be put in place urgently to prevent the growing number of fall-related mortality for men over 40 years old and women over 70 years old. Gains observed in the recent period, relative risks (RRs), and cohort RRs may be related to improved healthcare and better education.

## Background

Unintentional falls are a major global public health issue, which greatly affects health and medical care costs (1). In 2019 alone, unintentional falls caused around 752,536 deaths, representing 17.5% of all injury-related deaths worldwide (2). In addition, unintentional falls were responsible for 39,362,279 disability-adjusted life years around the world, which exceeded the total number of years of disability from transport injuries, drowning, burns, and poisoning (2). The severity of fatality due to unintentional falls varies in different regions. Low- and middle-income countries suffered more than 80% of unintentional fall-related deaths, while about 60% happened in the Western Pacific and Southeast Asia (3). However, current epidemiological and prevention studies are mainly confined to developed areas.

Unintentional falls also seriously threaten the lives and health of people in China. However, there are few studies on the assessment of unintentional fall mortality in China. The existing trend analyses of unintentional fall mortality in China were limited to a specific region (4, 5) or population, and their research period was relatively short (6) or outdated (7). The data in those studies were age-adjusted by the standard population of China, which is not conducive to comparing mortality between different countries. More importantly, the underlying causes of long-term trends have not been fully analyzed, and the relative risks caused by period and cohort effects are still unknown. To address the limitations of existing studies, this study aimed to examine the long-term trends in unintentional fall mortality in China from 1990 to 2019, investigating age-, period-, and cohort-specific effects using data from the Global Burden of Disease Study 2019 (GBD 2019) within the age–period–cohort (APC) framework.

## Methods

### Data Sources

Data were obtained from the GBD 2019. The GBD study provided comprehensive and internally consistent estimates of many health measurement indicators by sex, age, and region for each calendar year through international research collaboration. In GBD 2019, estimates of age-sex specific all-cause and cause-specific mortality from 369 diseases and injuries in 204 countries and territories from 1990 to 2019 were provided (2). Methods used in the GBD estimation process have been described extensively in previous publications (2, 8, 9). The original mortality data of the unintentional falls in China were from three primary data sources, i.e., Disease Surveillance Points, Maternal and Child Surveillance System, and Chinese Center for Disease Control and Prevention Cause of Death Reporting System (9). All deaths due to unintentional falls were identified according to the International Classification of Disease (W00–W19 in ICD-10, and E880–888 in ICD-9 excluding E887) (9). The data of unintentional fall-related deaths and corresponding population information in China were derived for this study. To describe the time trend, unintentional fall-related mortality rates of Chinese people were adjusted to the GBD global standard population using the direct method (2).

### Statistical Analysis

In this study, the APC model was used to analyze the trends in the three dimensions of age, period, and cohort of unintentional fall-related mortality risk in China. For the APC analysis, the mortality and population data for unintentional falls were sorted into continuous 5-year periods from 1990 to 2019 and consecutive 5-year age intervals from 0 to 84 years old. The exact age groups after the age of 85 years in China were not provided in the GBD database. The APC analysis was based on a log-linear model for the incidence rates with additive effects from age, calendar period, and birth cohort as shown in the formula below:


(1) $$\begin{array}{l} \rho_{pa}=\alpha_{a}+\beta_{p}+\gamma_{c} \end{array}$$

where ρ_*pa*_ is the expected incidence rate, while α_*a*_, β_*p*_, and γ_*c*_ indicate the effects of age, period, and cohort, respectively. In the model, the age effect represents the effect of changes in the outcome risk associated with different age groups; the period effect represents the effect that can affect the changes in the outcome risk of all age groups at different times simultaneously. The cohort effect represents the effect of changes in the outcome risk associated with groups born in the same birth period (10–12). The study of Holford proposed and proved that if age, period, and cohort trends are orthogonally decomposed into linear and non-linear parts, there are many beneficial functions that are constant and estimated (13, 14). This method is called the Estimable Function algorithm. This study mainly focused on the following estimable functions. Net drift represents the overall average annual percentage change of the logarithmic linear rate during a certain period. Local drifts represent the average annual percentage change of the logarithmic linear rate for each age group. The longitudinal age curve indicates the fitted longitudinal age-specific rates in the reference cohort adjusted for period deviations, which represents the age effect. The period relative risk (RR) indicates the ratio of the age-specific rate value in a certain period to that in the reference period, which represents the period effect; the cohort RR indicates the ratio of the age-specific rate value in a certain birth cohort to that in the reference cohort, which represents the cohort effect (11, 14).

In this study, the R package for APC analysis (14) developed by the Biostatistics Branch of National Institutes of Health, USA was used to model and obtain the above estimable functions. In the APC analysis, the central age group, period group, and birth cohort group were, respectively, defined as reference values. If the number of categories was even in the group, the reference value was set to the lower of the two central values (14). The Wald Chi-Square test was used to test whether the estimable functions were statistically significant. The R programming language version 3.6.3 (R Foundation for Statistical Computing, Vienna, Austria) was used to conduct all two-tailed statistical tests. *P*-values < 0.01 were regarded as statistically significant.

### Patient and Public Involvement

There has been no patient and/or public involvement in the study design, data collection, data analysis, and writing of this research.

## Results

**Figure 1:**
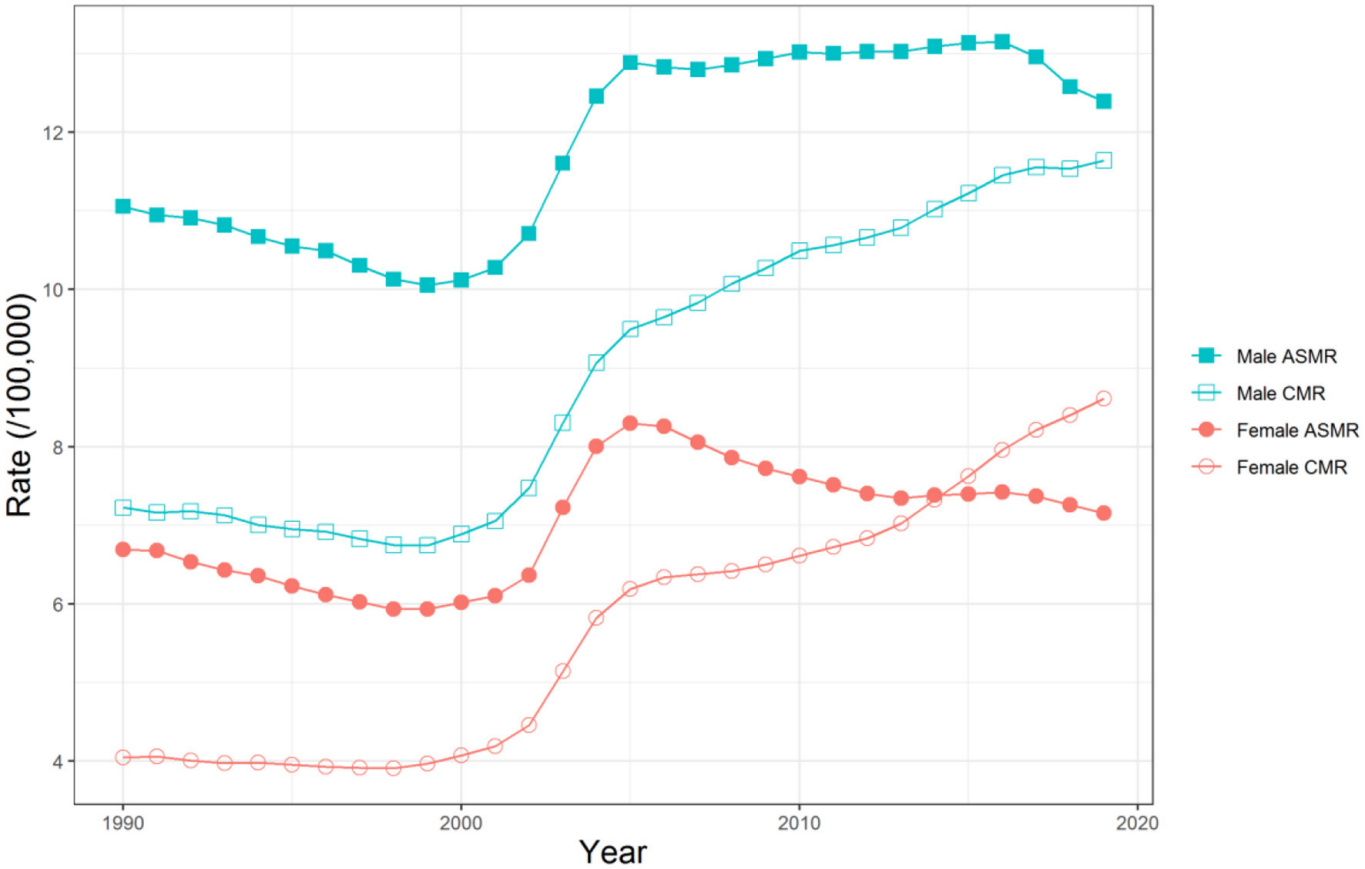
Trends of the crude mortality rates (CMR) and the age-standardized mortality rates (ASMR) due to unintentional falls for both sexes in China, 1990–2019.

In general, from 1990 to 2019, a total of 1,840,847 and 1,113,415 deaths due to unintentional falls were reported among 20,364,025,044 and 19,296,319,576 person-years at risk for Chinese men and women, respectively. Figure 1 shows the crude mortality rates (CMR) and the age-standardized mortality rates (ASMR) due to unintentional falls for both sexes in China from 1990 to 2019. Overall, although the CMR for both sexes showed a significant upward trend, the ASMR for both sexes only increased slightly. Specifically, the CMR for men and women decreased slightly before 1999, and then gradually increased to 11.64 per 100,000 and 8.61 per 100,000, respectively, with the fastest increase rates from 1999 to 2004. For ASMR, the mortality of men decreased before 1999, then increased rapidly to 2005, and decreased to 12.39 per 100,000 in 2019 after slight fluctuation. The mortality of women was similar to that of men but it steadily decreased to 7.16 per 100,000 after 2005. During this period, the risk of death from unintentional falls was higher in Chinese men than in women.

**Figure 2:**
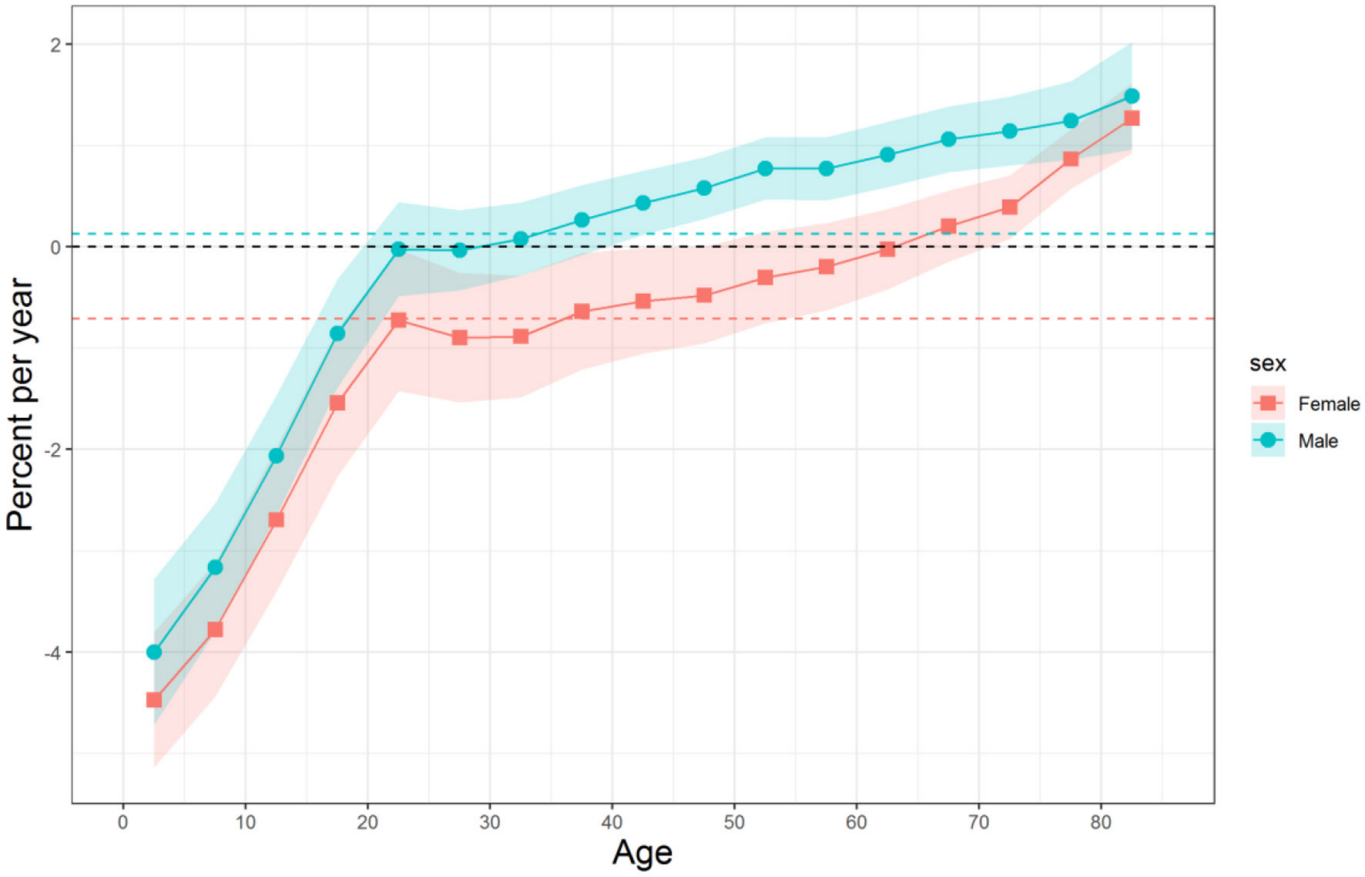
Local drifts with net drift of unintentional falls mortality for Chinese men and women. Age group-specific annual percent changes (%) and the corresponding 95% CIs. The overall annual percent changes (%) were indicated by the horizontal-colored dotted lines.

The net drift, which represents the overall annual percentage change, and local drifts, which indicate annual percentage changes for each age group, for Chinese men and women were shown in Figure 2, respectively. The net drift of unintentional falls mortality was 0.13% (95% CI, −0.04 to 0.3%) per year for men and −0.71% (95% CI, −0.96 to −0.46%) per year for women. The local drift values of unintentional falls mortality for both sexes increased with age group; the rising trend was fast at first and began to slow down after 20 years old. It should be noted that the local drift values were negative in the age groups of 0–19 years old for men and that of 0–49 years old for women, whereas they were positive for men over 40 years old and for women over 70 years old. Detailed data on the local drift values for unintentional falls mortality is displayed in Supplementary Tables S1, S2.

**Figure 3:**
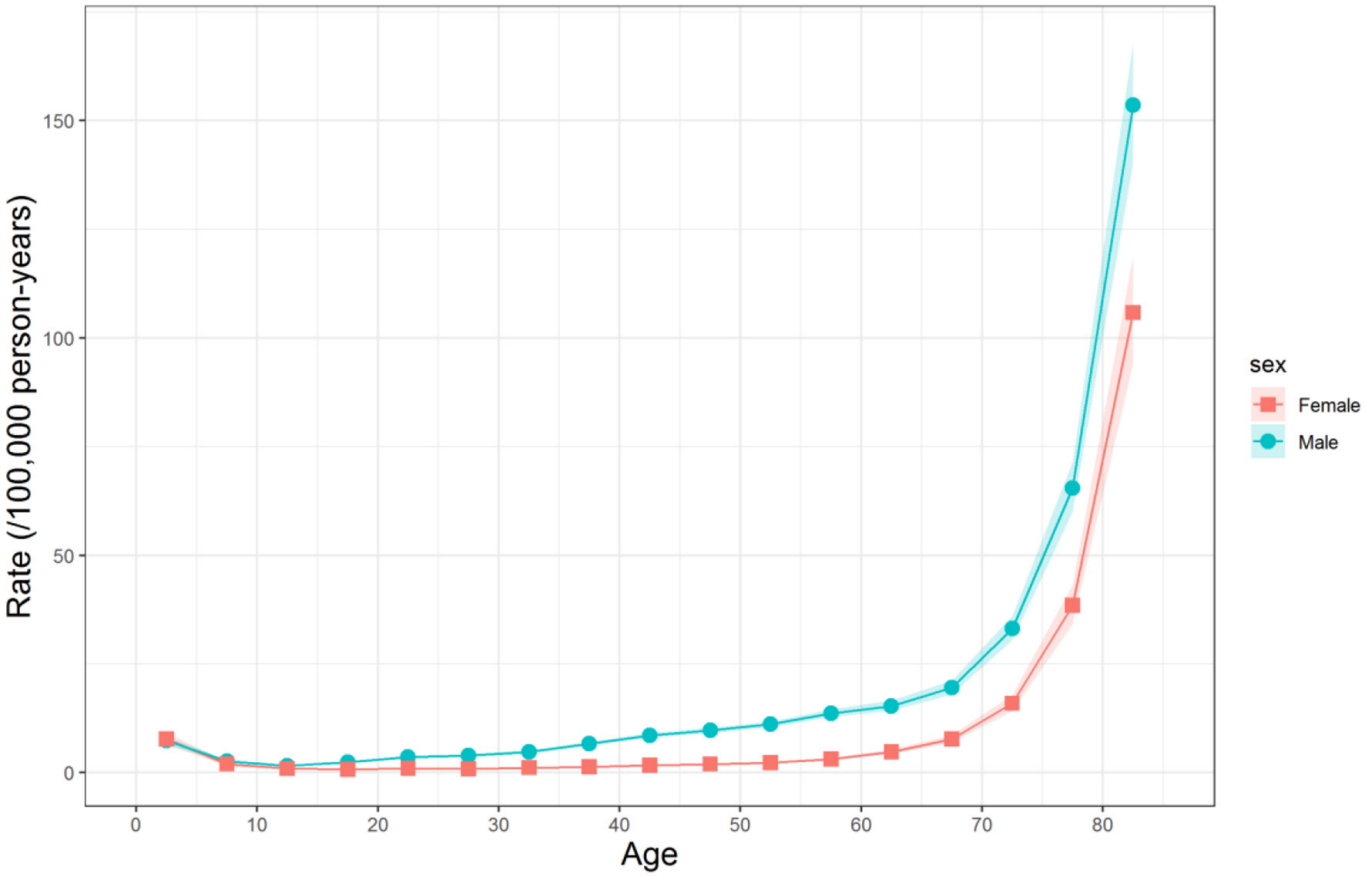
Longitudinal age curves of unintentional falls mortality for Chinese men and women. Fitted longitudinal age-specific rates and the corresponding 95% CIs.

The longitudinal age curves of unintentional falls mortality for Chinese men and women are displayed in Figure 3. Overall, for individuals born in the same cohort, the mortality risk of unintentional falls decreased first and then increased constantly with age for both sexes, and the rising speed became faster in the old age stage. Specifically, the mortality risk decreased to the bottom in the age group of 10–14 years old for men and 15–19 years old for women and then began to rise slowly. After 60 years old, the mortality risk for both sexes increased rapidly. In general, the risk of death from unintentional falls was higher in Chinese men than in women in the whole life stage.

**Figure 4:**
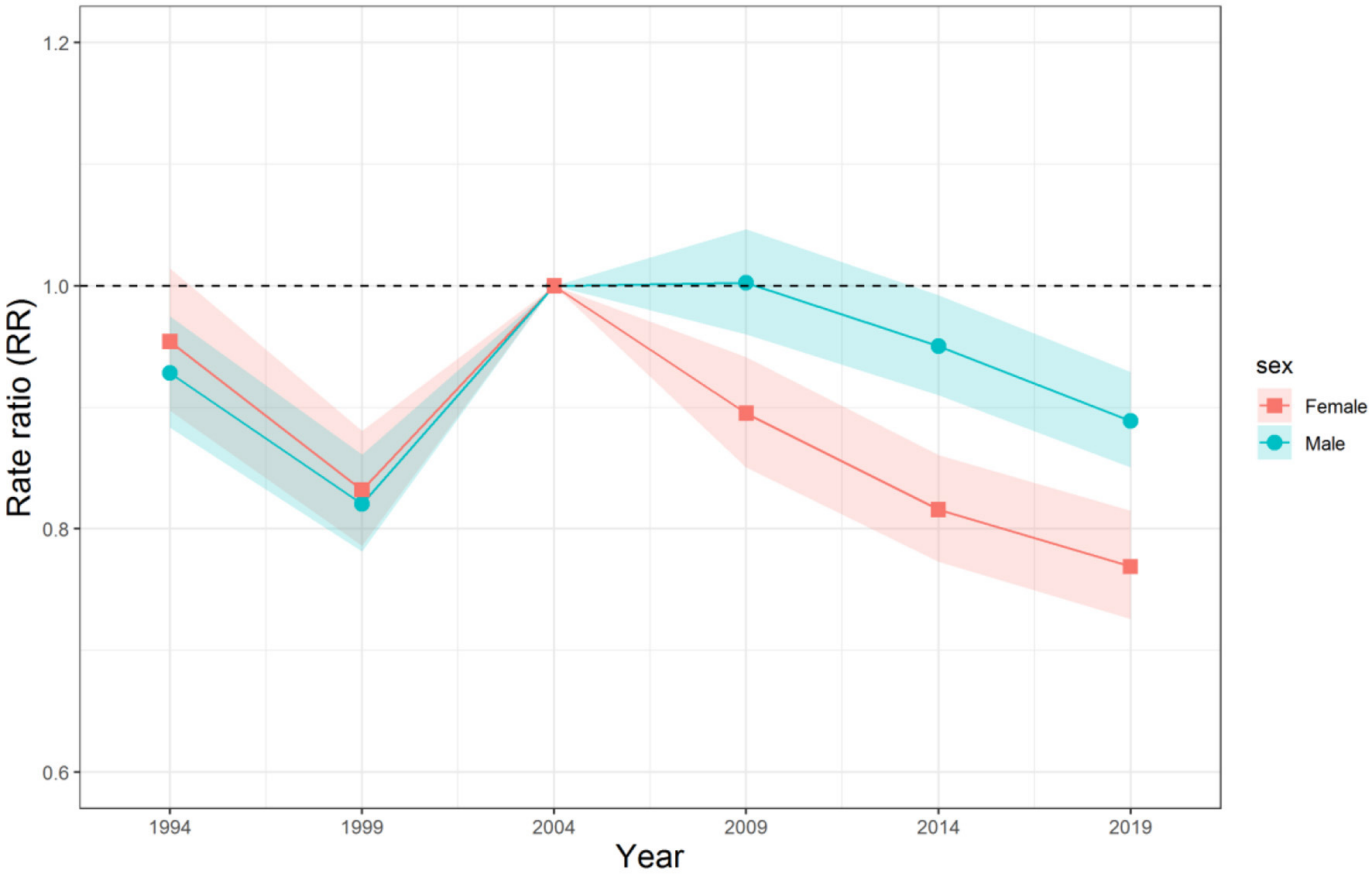
Period relative risks (RRs) of unintentional falls mortality for Chinese men and women. The relative risk of each period to the 2004 reference year and the corresponding 95% confidence intervals.

The estimated period RRs of mortality due to unintentional falls for Chinese men and women are shown in Figure 4. The period RRs for both sexes decreased for most of the period, except for the increase between 1999 and 2004. It can be seen that the period RRs for mortality risk of men and women showed a similar downward trend before 1999, and then rose from 1999 to 2004. After that, the period RR for men was flat and began to decrease again after 2009, whereas the period RR for women began to decrease again after 2004. Overall, the period RR for mortality risk of women reduced more than that of men over the study period.

**Figure 5:**
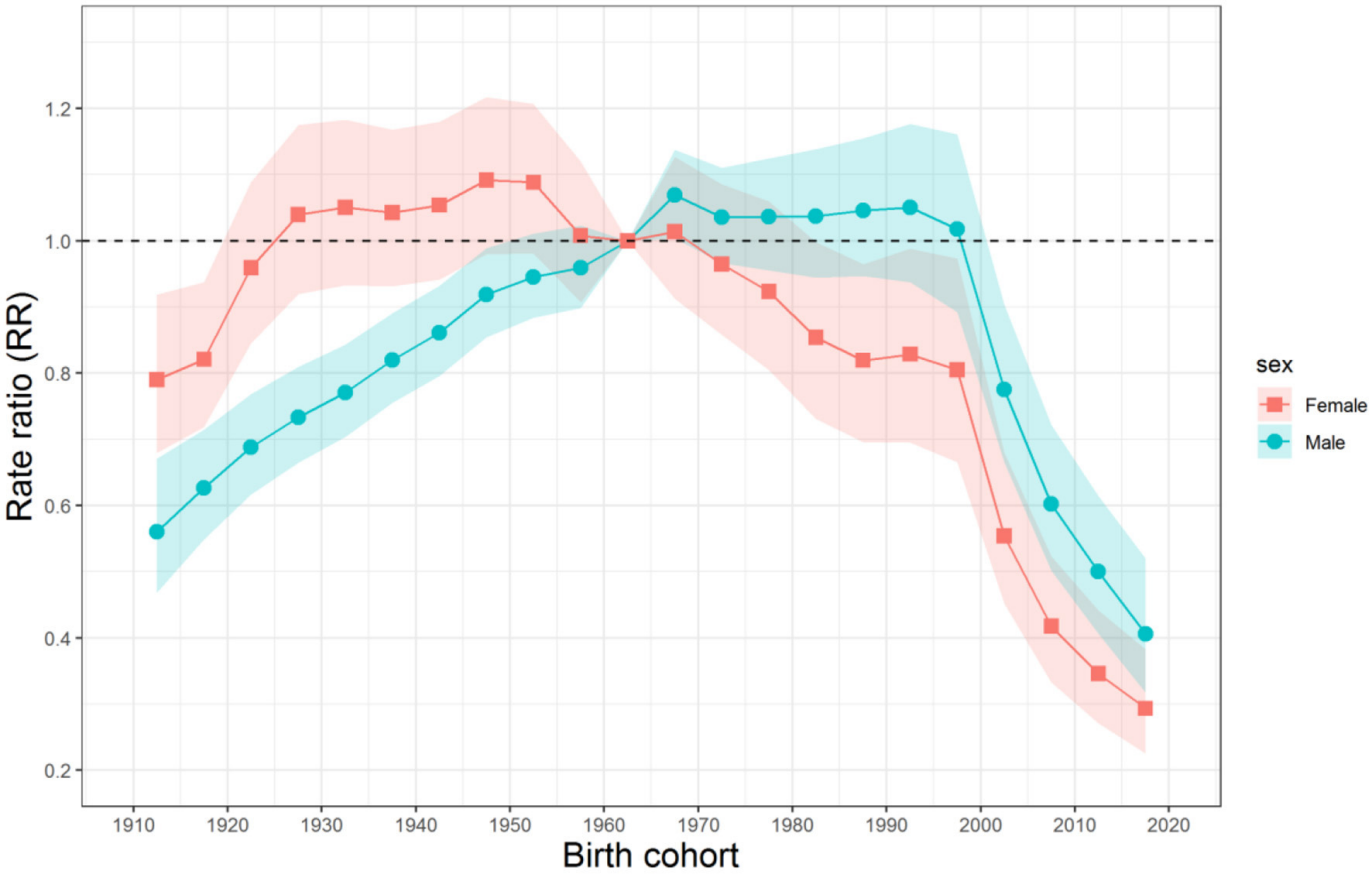
Cohort RRs of unintentional falls mortality for Chinese men and women. The relative risk of each cohort to the 1960–1964 reference cohort and the corresponding 95% CIs.

The estimated cohort RRs of mortality due to unintentional falls for Chinese men and women are displayed in Figure 5. The cohort RRs showed a rapidly decreasing trend after the cohort 1990–1994 for both sexes. Before this cohort, the cohort RR for men increased and then fluctuated slightly from the cohort 1960–1964 to 1995–1999; for women, it generally increased and then decreased after the cohort 1945–1949. Overall, the cohort RR for mortality risk of women reduced more than that of men over the study period. In addition, Wald test results showed that the cohort and period RRs of unintentional falls mortality for both sexes were statistically significant (*P* < 0.01 for all), as well as the net drift and local drifts (*P* < 0.01 for all).

## Discussion

As far as we know, this was the first reported study to examine the long-term trends of sex-specific and age-specific mortality due to unintentional falls in China and to investigate its age, period, and cohort effects within the APC framework. It can be found that the ASMRs of unintentional falls fluctuated to 12.39 and 7.16 per 100,000 in 2019 for men and women, respectively, and the net drift of unintentional fall mortality was 0.13% (95% CI, −0.04 to 0.3%) per year for men and −0.71% (95% CI, −0.96 to −0.46%) per year for women from 1990 to 2019. During this period, the risk of death from unintentional falls was higher in Chinese men than in women. It should be noted that the local drift values were negative in the age groups of 0–19 years old for men and that of 0–49 years old for women, whereas they were positive for men over 40 years old and for women over 70 years old. Moreover, our results indicated that there are significant age, cohort, and period effects in the mortality trends of unintentional falls.

Although unintentional falls and deaths can occur in all age groups, age is still recognized as the most critical risk factor for fall-related fatality (15). According to the longitudinal age curve results, it can be seen that the mortality risk of unintentional falls decreased first and then increased constantly with age for both sexes, and the rising speed became faster in the old age stage. The risk of death from unintentional falls was higher in Chinese men than in women in the whole life stage. In the life stage of 0–14 years, the most at-risk age group of unintentional falls death in Chinese men and women is 0–4 years old, and the risk of that decreased in the following 5–9 and 10–14 years old. This was mainly due to the weaker motor balance ability, risk response-ability, and emergency perception ability of children aged 0–4 years old compared with older ones (16). In addition, incomplete skull development of a toddler, more substantial innate curiosity, and higher center of gravity in activities may also be important reasons for the higher risk of unintentional falls death (17–19). As a high-risk group, children suffered falls mainly due to improvement of independence, accompanied by more challenging behaviors and less adult supervision (5, 20). Relevant studies had shown that unintentional fall-related deaths of children aged 0–14 mainly occurred from a height (falls from one level to another) (17, 21), and many children's fall injuries could be prevented by simple safety measures, using protective devices or avoiding the use of specific devices (22). Many developed countries have carried out studies on prevention of children's falls for a long time. Some of the findings have been translated into prevention practices, which were considered to achieve desirable effects after evaluation, e.g., the “Children Can't Fly” program (23). At present, there are few epidemiological studies on the falls of children in China, and there are even fewer prevention projects (17). More related research and evaluation of preventive measures on the falls of children are needed in China.

The mortality risk of unintentional falls increased gradually with age in the life stage of 15–59 years old for men and 20–59 years old for women and then increased rapidly after the age of 60 for both sexes. The elderly [over 60 years old (24, 25), or over 65 years old (21)] who died of unintentional falls mainly occurred on the same high level, resulting from slips and trips. Existing studies had thoroughly studied the risk factors of unintentional falls in the elderly. It was generally believed that the high mortality risk of unintentional falls for the elderly was mainly related to their physical condition, psychological condition, disease status, medication factors, and behavior and environmental factors (26). It could be easily concluded that, compared with the physical condition and disease status, the other related factors could be intervened and changed. Therefore, prevention, which requires the close cooperation of the elderly, their families, and society, is the key to reduce unintentional falls (27). There is evidence showing that (28) physical exercise, rehabilitation treatment, medication management, and vitamin D deficiency treatment were very effective single intervention measures to prevent fall-related fatality for the elderly. To curb the trend of rising mortality from unintentional falls among the elderly, it is urgent to take such intervention measures.

The impact of the ICD coding issue should be first considered when it comes to the period effect of mortality risk (11). In China, the improvement of the cause-of-death reporting may be closely related to the rise of the period RRs of mortality due to unintentional falls. This is because when the reporting quality of the cause-of-death code was not desirable in the past, fall-related deaths were likely to be underestimated. For example, an elderly person who has suffered a fall injury such as a rib fracture was likely to die of pneumonia or other complications several weeks or months later, but the doctor responsible for reporting the cause of death may ignore the fall injury and filled the root cause of death as pneumonia or other deadly diseases. The specific analysis was reflected in a study on the reporting of causes of death in injury and poisoning in China (29) and a subgroup analysis of fall deaths in the United States (30). In addition, the following factors may also play a role according to the available data (31). On the one hand, the rising prevalence of chronic diseases, the increased medication, and the prolonged life expectancy in China would increase the risk of fatal falls, especially for the middle-aged and the elderly. On the other hand, with the rapid urbanization in China, the living environment of people (higher floors, more solid grounds, etc.) has become more prone to falls, leading to higher period RRs.

The reason for the decline in the period RRs of mortality due to unintentional falls in the Chinese population may be related to the improvement of medical treatment. In many cases, falls did not end in death immediately on the spot (21), so whether the health care provider can seize the golden rescue period for timely rescue of the injured is highly associated with mortality to a great extent. In China, various scales of first-aid centers have been established at and above the county level, which shortens the period from the fall occurrence to treatment [the “golden hour” (32)], thus improving the survival rate. In addition, during the study period, the introduction and promotion of new diagnostic tools, e.g., CT and MRI systems, the standardized treatment and protocols for trauma patients, and better treatment technology, e.g., fracture treatment technology and application of bone material, among others., and treatment conditions (more ICU wards), could all improve the survival rate of the people who suffered unintentional falls (33), thus providing a positive impact on the period RRs.

The declining trend of the cohort RRs of mortality due to unintentional falls for both sexes in the younger birth cohort was likely to be related to higher body mass index (BMI) and better education. There is evidence showing that low BMI is a critical risk factor for unintentional fall-related fatality, because of the association between BMI and bone mineral density on fall-related fracture risk (34). Relevant studies showed that the younger birth cohort in China had a higher BMI than the older birth cohort (35). In addition, lower education background is also considered as one of the risk factors for unintentional fall-related fatality. This is because falls could be prevented largely, and people with better education backgrounds are more likely to effectively prevent falling injury after receiving the same relevant safety education (34). Overall, the younger birth cohort in China had a higher education background than the older birth cohort, so it had a lower risk of unintentional fall-related fatality.

Compared with other birth cohorts, the male birth cohort born around 1965–1999 had the highest cohort RRs of mortality due to unintentional falls. This may be related to the occupational risk of the male group. Unintentional falls for adults can be divided into occupational and non-occupational. The occupational falls mainly occur in construction workers, followed by manufacturing, transportation, and storage workers (36). Almost all the workers in these industries were male in China. In the past three decades, China has witnessed rapid development in construction, manufacturing, transportation, storage, and many other industries (37). According to the appropriate working-age population, the main body of workers in these industries were male cohorts born around 1965–1999, leading to the highest cohort RRs in these birth cohorts.

There were several limitations to this study. First, the completeness and accuracy of unintentional fall-related mortality data in China might cause bias in some way, although there were effective corrective measurements, including the incompleteness, under-reporting, and misclassification corrections, as well as the redistribution of the garbage code, to improve data quality and comparability in the GBD study (2, 8). However, most would agree that the possible bias in the present study has been greatly reduced compared with the study using unadjusted raw data. Since the integrity and accuracy of GBD mortality data in China have not been reported yet, it should be necessary to conduct relevant research in the future. Secondly, because the original data of death cause in China could only be obtained on the 5-year-old age group, and the age and period intervals in the APC model should be fixed and equal, this APC analysis has to be carried out by the 5-year intervals (14). Some subtle changes might have been eliminated. When more minor age group data is available in the future, more precise research should be carried out in smaller units. Third, an ecological fallacy was possible since the interpretation of results from the population level in APC analysis did not necessarily apply to individuals (12). Therefore, the relevant hypotheses of the study still need to be further confirmed in future individual-based research.

We concluded that unintentional falls are still a major public health problem that disproportionately threaten the lives of men and women in China. Since fall-related mortality increased for men over 40 years old and women over 70 years old, it should be urgent to implement targeted interventions. Improvements were concentrated in the age groups of 0–19 years old for men and 0–49 years old for women. The mortality trend of unintentional falls in China had significant age, period, and cohort effects. Gains observed in recent period RRs and cohort RRs may be related to improved healthcare and better education.

## Code Availability

The codes used during and/or analyzed during the current study are available in the Github repository, [https://github.com/CBIIT/nci-webtools-dceg-age-period-cohort/blob/master/apc/apc.R/].

## Data Availability Statement

Publicly available datasets were analyzed in this study. This data can be found here: http://ghdx.healthdata.org.

## Author Contributions

ZW and FP designed research and wrote the paper. ZW, YH, and FP performed research, analyzed data, reviewed all paper drafts, and gave approval to the final version. All authors contributed to the article and approved the submitted version.

## Funding

This study was supported by the National Natural Science Foundation of China (No. 81903396). The findings and conclusions contained within are those of the authors and do not necessarily reflect positions or policies of the related funding.

## Conflict of Interest

The authors declare that the research was conducted in the absence of any commercial or financial relationships that could be construed as a potential conflict of interest.

## Publisher's Note

All claims expressed in this article are solely those of the authors and do not necessarily represent those of their affiliated organizations, or those of the publisher, the editors and the reviewers. Any product that may be evaluated in this article, or claim that may be made by its manufacturer, is not guaranteed or endorsed by the publisher.
