# Supplementary material for: Long-Term Trends in Unintentional Fall Mortality in China: A Population-Based Age-Period-Cohort Study
Source: Front Public Health. 2021 Nov 24;9:749295. doi: 10.3389/fpubh.2021.749295 (PMC8744467; doi:10.3389/fpubh.2021.749295)
Supplement: Supplementary file 1 [file Data_Sheet_1.docx]

**Long-term trends in unintentional falls mortality in China: a population-based age-period-cohort study**

**Zhenkun Wang, PhD^1,2^, Youzhen Hu, BS^1,3^, Fang Peng, PhD^1^***

1 Outpatient Department, Tongji Hospital, Tongji Medical College, Huazhong University of Science and Technology, Wuhan, Hubei, 430030, China

2 Department of Scientific Research, Tongji Hospital, Tongji Medical College, Huazhong University of Science and Technology, Wuhan, 430030, China

3 Department of Emergency, Tongji Hospital, Tongji Medical College, Huazhong University of Science and Technology, Wuhan, 430030, China

### Correspondence

Peng Fang, PhD

Outpatient Department, Tongji Hospital, Tongji Medical College, Huazhong University of Science and Technology, Wuhan, Hubei, 430030, China

Email: pengfang@tjh.tjmu.edu.cn

Address: No.1095 Jiefang Road, Wuhan, Hubei, 430030, China

Tel/Fax: +86-027-8366-2871

**Appendix A. Supplementary data**

**Table S1** Local drifts of unintentional falls mortality for specific age groups in Chinese men
**Table S2** Local drifts of unintentional falls mortality for specific age groups in Chinese women

**Table S1** Local drifts of unintentional falls mortality for specific age groups in Chinese men

| **Age** | **Percent per Year** | **CILo** | **CIHi** |
| --- | --- | --- | --- |
| 0-4 | -3.9999 | -4.7159 | -3.2784 |
| 5-9 | -3.1666 | -3.7974 | -2.5317 |
| 10-14 | -2.0625 | -2.656 | -1.4653 |
| 15-19 | -0.855 | -1.3873 | -0.3198 |
| 20-24 | -0.0225 | -0.4857 | 0.4429 |
| 25-29 | -0.0331 | -0.428 | 0.3634 |
| 30-34 | 0.0763 | -0.2844 | 0.4382 |
| 35-39 | 0.2671 | -0.0772 | 0.6125 |
| 40-44 | 0.4341 | 0.117 | 0.7523 |
| 45-49 | 0.5788 | 0.2756 | 0.8828 |
| 50-54 | 0.7738 | 0.4675 | 1.081 |
| 55-59 | 0.7705 | 0.457 | 1.0849 |
| 60-64 | 0.9111 | 0.5898 | 1.2334 |
| 65-69 | 1.0605 | 0.735 | 1.3869 |
| 70-74 | 1.1439 | 0.8055 | 1.4835 |
| 75-79 | 1.2461 | 0.8603 | 1.6335 |
| 80-84 | 1.4902 | 0.9616 | 2.0216 |

**Table S2** Local drifts of unintentional falls mortality for specific age groups in Chinese women

| **Age** | **Percent per Year** | **CILo** | **CIHi** |
| --- | --- | --- | --- |
| 0-4 | -4.473 | -5.139 | -3.8024 |
| 5-9 | -3.7775 | -4.4378 | -3.1126 |
| 10-14 | -2.6955 | -3.4054 | -1.9803 |
| 15-19 | -1.5421 | -2.2669 | -0.812 |
| 20-24 | -0.7246 | -1.4228 | -0.0215 |
| 25-29 | -0.8976 | -1.5359 | -0.2552 |
| 30-34 | -0.8847 | -1.4849 | -0.2808 |
| 35-39 | -0.6363 | -1.2105 | -0.0587 |
| 40-44 | -0.5348 | -1.0542 | -0.0128 |
| 45-49 | -0.4781 | -0.9534 | -0.0005 |
| 50-54 | -0.304 | -0.7556 | 0.1497 |
| 55-59 | -0.1978 | -0.6264 | 0.2326 |
| 60-64 | -0.024 | -0.4191 | 0.3727 |
| 65-69 | 0.204 | -0.1459 | 0.5551 |
| 70-74 | 0.3909 | 0.0778 | 0.705 |
| 75-79 | 0.8674 | 0.5745 | 1.161 |
| 80-84 | 1.269 | 0.9234 | 1.6158 |
